# Editorial: Biomolecule production in plant synthetic biology

**DOI:** 10.3389/fpls.2026.1857952

**Published:** 2026-05-01

**Authors:** Sichul Lee, Moonhyuk Kwon

**Affiliations:** 1 Department of Agricultural Biology, National Institute of Agricultural Sciences, Rural Development Administration, Jeonju, Republic of Korea; 2 Division of Life Science (BK21 Four), Anti-aging Biomaterial Cell-factory Regional Leading Research Center, Research Institute of Molecular Alchemy, Gyeongsang National University, Jinju, Republic of Korea

**Keywords:** biomolecule, plant, production, sustainability, synthetic biology

In the last few decades, synthetic biology research has been mainly performed with microbes. In contrast, plant researchers have traditionally focused on the physiology, growth, resistance, and natural products based on the molecular biology and genetics techniques. However, emerging technologies are redefining plants as a new platform for producing specific target compounds. Accordingly, the application of plants is expanding into the synthetic biology field through the application of design, control, and biosynthetic systems to engineer plants. In this context, the Research Topic entitled *“Biomolecule Production in Plant Synthetic Biology”* presents emerging studies that, based on these new research directions and technologies, aim to industrially produce target molecules through the optimization of biological components, including molecular parts, and the redesign of metabolic pathways to develop and enhance heterologous plant platforms.

Park et al. provide insights into core research methods in plant synthetic biology, such as biosynthetic circuits design, genome editing, and *de novo* DNA synthesis. In particular, plant biotechnology developments shift the paradigm from simple gene-level regulation to the redesign of metabolic networks for the production of useful materials at the industry level, leading to a conversion in research structure and direction. Hereby, the potential for sustainable biomaterial production is infinite using plants as a chassis in synthetic biology. However, improvements of the various limits in plant biotechnology (low transformation efficiency, unstable metabolic flux following multiplex gene introduction, regulation of genetically modified plants, biosafety, and standardization for industrial uses) must come first. Therefore, the improvement of gene transfer techniques, the development of predictive calculating models, and flexible deregulations are necessary for the application of a plant-based biomanufacturing system in industry.

In plant synthetic biology, it is one of the most critical aspects to establish a stable metabolic pathway through the selection and optimization of molecular parts performance. From Lee et al.’s review, it can be seen how catalyst efficiency, substrate specificity, intracellular localization, reaction rate regulation, and metabolic engineering affect the synthetic biological production of the flavonoids. Diosmin is a natural flavonoid extracted from citrus fruits that acts as a vascular strengthening agent, increasing blood vessel elasticity and reducing inflammation. It is primarily used to alleviate symptoms of hemorrhoids, varicose veins, leg edema, and venous insufficiency, and helps improve blood circulation by increasing venous tone. To produce the diosmin in tobacco, Lee et al. modularly reconstructed the 10-step biosynthetic pathway and transiently expressed it in *Nicotiana benthamiana* leaves without supplying additional precursors, resulting in the first production of diosmin in a heterologous plant system with 37.7 μg/g FW productivity. This study shows the possibility of replacing the active pharmaceutical ingredient (API) source from chemical synthesis to a synthetic biological approach, a sustainable and eco-friendly alternative system.

In another Lee et al.’s advanced research, the chrysoeriol biosynthetic pathway was artificially reduced from the native 8-step pathway to 4-steps by the expression of 5 genes, successfully producing the target molecule and improving the anti-oxidant activity in plant tissue. This study also highlighted the transient metabolic gene expression as a rapid and efficient approach using transient agroinfiltration for metabolites prototyping and short-term production, avoiding unnecessary time and cost-consuming for the development of stable transgenic plants.

Besides small molecules, Kim et al. showed that the recombinant protein production in plants is also a considerable alternative to animal cell-based production. A decomposable vector based on geminivirus derived from TYLCV, HYVV, BMCTV enabled a rapid, high-quantity protein accumulation in *N. benthamiana*. In the presence of a gene-silencing suppressor, the protein yield reached up to 12% of the total soluble protein. This result suggested the competitive potential of the viral expression platform for molecular farming in fields where a rapid and scalable production system is essential, such as vaccine and API production.

Meanwhile, Aschern et al. developed a new platform (MolnClo) by inserting an intron according to the MoClo standard increased the expression level of luminescence protein and functional enzymes. This platform supplemented the shortage of stable gene expression in *Chlamydomonas reinhardtii*, accordingly, it expanded metabolic engineering tools to the microalgae-based synthetic biology field by providing an improvement of gene expression. As a consequence, this increases the potential for utilizing algae systems as a low-cost bioproduction platform suitable for large-scale bioreactor applications and closed-system cultivation.

From the articles in this Research Topic, predictable and expandable plant synthetic biology is the core technology in bio-manufacturing. Particularly, it demonstrates that technologies previously difficult to realize can be implemented through host-specific expression techniques, modular metabolic pathway reconfiguration, and the optimal combination and fusion of bio-components. This conversion will be stimulated through high-throughput screening using an automated system, the application of AI to predict and design enzyme activity and metabolic pathways, deregulation for commercialization, the selection of plants optimized to target materials, and the expansion of chassis to photosynthetic single-cell organisms.

Overall, from the recent advanced researches, we can affirm the big potential of the plant and microalgae as the core platform in the synthetic biology field for the industrially valuable biomaterials production, such as API and functional foods. Furthermore, expansion to agriculture and bio-manufacturing from the continuous advances of plant synthetic biology technology and its demonstration research can redefine the bio-based production using the photosynthetic organism as a complementary host of microbes in the bio-economy.

